# Political dynamics promoting the incremental regulation of secondhand smoke: a case study of New South Wales, Australia

**DOI:** 10.1186/1471-2458-6-192

**Published:** 2006-07-21

**Authors:** Katherine Bryan-Jones, Simon Chapman

**Affiliations:** 1 School of Public Health, University of Sydney, New South Wales 2006, Australia

## Abstract

**Background:**

The history of governmental responses to the accumulation of scientific evidence about the harms of secondhand smoke (SHS) presents an intriguing case study of incremental public health policy development. Australia has long been considered a world-leader in progressive tobacco control policies, but in the last decade has fallen behind other jurisdictions in introducing SHS legislation that protects all workers. Bars, clubs and pubs remain the only public indoor spaces where smoking is legally permitted, despite SHS exposure in the hospitality industry being higher and affecting more people than in any other setting after domestic exposure. This paper examines the political dynamics that have shaped this incremental approach to SHS.

**Methods:**

In-depth interviews with 21 key stakeholders in the state of New South Wales (NSW), including politicians, their advisors, health officials and tobacco control advocates, were conducted and subjected to thematic content analysis. Interviewees' comments provided insights into the dynamics surrounding the debates and outcomes of SHS legislative attempts and the current political environment, and about how to progress SHS legislation.

**Results:**

SHS restrictions have been delayed by several broad factors: the influence of industry groups successfully opposing regulation; issue wear-out; and political perceptions that there is not a salient constituency demanding that smoking be banned in bars and clubs. Interviewees also provided suggestions of strategies that advocates might utilise to best overcome the current political inertia of incremental compromises and achieve timely comprehensive smoking bans.

**Conclusion:**

Advocates concerned to shorten the duration of incremental endgames must continue to insist that governments address SHS fundamentally as a health issue rather than making political concessions to industry groups, and should broaden and amplify community voices calling on governments to finish the job. Publicity to the growing number of state and national governments that have successfully implemented total bans over the past decade is likely to make incrementalism an increasingly unattractive political option.

## Background

The history of government response to the emerging evidence on the harmfulness of secondhand smoke (SHS) typically presents intriguing case studies of incremental public health policy, often spanning decades. By incremental, we mean a process whereby governments gradually introduce restrictions on smoking in particular settings rather than acting to simultaneously introduce smokefree requirements in all enclosed public places where a duty-of-care toward employees, patrons or the visiting public can be demonstrated to exist. Incremental policy acknowledges that SHS should be controlled because of its health consequences, but effectively gives priority to protecting workers and citizens in certain contexts while permitting exposure to continue in others. The most usual pattern features the introduction of smokefree public transport, followed by office and indoor workplace restrictions, then smokefree restaurants and finally bars (pubs, taverns) and gambling venues. Incremental policy is not predicated on arguments that exposure in the areas of priority implementation is more severe or affects more people and is thus deserving of more immediate control than in the later regulated areas. Indeed, exposures in bars are among the highest recorded [1]. Instead, other cultural, economic and ideological factors are publicly invoked by policy makers and legislators to justify the delays [2].

SHS policy, while typically implemented by health authorities, is thus not "rolled out" according to rational risk reduction priorities, but as a form of regulatory paradox where those least severely exposed are the first to be protected, while those most exposed (bar and gambling venue workers) are the last. This incrementalism has been ridiculed as "half pregnant" policy [3] reflecting neo-Dickensian occupational health values whereby bar and casino staff are denied legal protections that are enshrined in law for all other indoor workers, restaurant staff and patrons, the relatively transient passengers of public transport and even elevator passengers.

In this paper we examine the recent history of incremental legislation to introduce smoke-free indoor environments in the most populous Australian state of New South Wales (NSW). Australia has one of the world's most advanced tobacco control programs [4,5] and has seen smoking prevalence fall lower than any other comparable nation, with daily smoking in those aged 14 and over now being 17.4% nationally, and 16.5% in NSW [6]. In Australia, legislative and regulatory power to control smoking in workplaces, public places and on public transport lies with the six state and two territory governments. The federal (national) government can control smoking only on federally controlled areas, such as airports and airlines. Almost all public policy debates on SHS have therefore occurred at the state level. A comprehensive outline of NSW and Australian SHS policies can be found in our detailed report [7].

In NSW, restrictions on smoking were first introduced on public transport in 1976 because of concerns about passenger comfort, and in cinemas and public halls in 1977 because of concerns about fire hazards (see Table 1). Public debate and policy change premised on SHS being a health risk to non-smokers did not commence until the 1980s.

The 1981 publication of Hirayama's Japanese study [8] on lung cancer in non-smoking wives of smokers caused a watershed in public policy about public tobacco use. Its publication saw a rapid escalation in scientific attention to the potential dangers of exposure to SHS. Australia's peak health and medical advisory body, the National Health and Medical Research Council, was one of the first of its kind in 1986 to review the accumulating evidence and recommend controls on smoking [9]. By 1987, passive smoking had become the leading topic receiving news coverage in all reportage on smoking matters [10], and remains so today [11].

Landmark events and reports generating widespread news coverage in the past two decades included legal actions brought by workers exposed to SHS, a successful but protracted court case brought against the Tobacco Institute of Australia for publishing a misleading statement about SHS [12] and debate surrounding attempts to introduce smokefree workplaces and bans on smoking on airlines.

After several failed legislative attempts (via private members' bills) to ban smoking in public places and workplaces in the early and mid-1990s, the NSW government passed the *Smokefree Environment Act 2000*, in time for the Sydney Olympics, banning smoking in enclosed public spaces, including restaurants and the dining areas of pubs and clubs. This law met little resistance, and was popular with the public [13].

Since at least 2001, NSW senior politicians have been quoted as saying that smoking bans in all workplaces are "inevitable" [14]. However, unlike the recent responses of the Irish, Norwegian, British and New Zealand governments to ban smoking in all public places, the NSW government has not responded to this "inevitability" with timely and comprehensive legislation; it instead continues to effectively exempt bars and clubs from a total ban. In early 2005, a final date for a complete indoor smoking ban in bars and licensed clubs was set for adoption in July 2007, although intense lobbying from the hospitality industry resulted in the government adopting a definition of an "enclosed space" that will allow smoking in areas that are up to 75% enclosed [15].

The pattern of bars being the "last bastion" to be addressed in smokefree air legislation appears to have no exceptions anywhere in the world where smokefree legislation has been introduced. The protracted paradox of those most exposed being the least protected, together with interest in how the hospitality industry – often with backing from the tobacco industry [16] – has succeeded in resisting smoke bans in spite of the risks posed particularly to its staff, makes this a compelling subject for study.

With each year that bars avoid smokefree legislation, further evidence accumulates about indoor air quality in bars [17], bar workers' SHS exposure [1,18] and their improved respiratory health when their workplaces go smokefree [19]. Similarly, as the community's experience of smokefree workplaces, restaurants and public transport becomes ever more routine, public support for smoking bans to be extended to bars also increases[20]. A large body of research has also accumulated showing either positive or negligible economic impacts on bar earnings following bans [21].

Notwithstanding this accumulating evidence, interest groups seeking to prevent and delay the introduction of smokefree bars have often succeeded in their objectives [22-27]. In Australia, the tobacco industry has a long history of attempting to discredit studies showing the harms of SHS [28] or circumvent smoking bans [29]. The *Australian Hotels Association *(AHA) and *Clubs NSW *have now replaced the tobacco industry as the dominant public voice opposing smoking bans in Australia [2]. Having acknowledged direct support from the tobacco industry, the AHA's opposition to bans is a classic example of the tobacco industry's "third party strategy" in action [30].

This paper examines the factors that key stakeholders in NSW believe characterise the policy development process of SHS legislation, and provides suggestions of what strategies might best accelerate the current political inertia of incrementally addressing the issue of smoking in bars and clubs.

## Methods

Data were gathered from interviews with key stakeholders who played a central role in or could provide insight into the development of various SHS restrictions in NSW over the past two decades. A total of 29 key individuals or organisations were invited to participate in this study, resulting in 21 participants. In-depth semi-structured interviews were conducted between February and April 2003 and each lasted between 1–1.5 hours. Participants included a cross-section of current and former politicians (6), political advisors (2), current and former senior health bureaucrats (5), tobacco control advocates (6), and catering industry (1) and labour union (1) representatives. Participants have been classified by their current or most recent affiliation and have been de-identified for confidentiality reasons.

Interviewees were asked to comment on their role in the development or delaying of SHS legislation in NSW, to reflect on the dynamics surrounding the debates and outcomes of legislative attempts and to provide suggestions about what they believed might progress comprehensive SHS legislation on the political agenda today. All participants were given the opportunity to review and approve the transcripts from the interviews. De-identified transcripts are available upon request from the first author.

The first author conducted a primary content analysis of the interviews [31]. The second author read the transcripts and discussed the coding and themes with the first author to reach consensus. The authors also searched media articles, press releases, NSW Parliamentary Hansard, journal articles and personal archives. These documents provided context, history and background to SHS legislative attempts in NSW, corroborated interviewees' comments and gave voice to individuals or groups who did not participate in this study. Quotations were selected as representative of themes that emerged in the process of analysis. This study was approved by the University of Sydney Human Ethics Research Committee.

## Results

### Inevitability and incremental advances

"I think most analysts would see that it's almost inevitable that it will finally happen, but the intriguing question is, what are the forces that are making it happen so slowly?" [health advocate]

Interestingly, despite suggestions that SHS was "relatively unimportant" [bureaucrat] and had "never been a big issue" [politician] on any government's political agenda, a political advisor reflected that there were few issues that sparked as much political, media and community interest as the debate over smoking. Nearly every participant agreed that the "writing is on the wall" [political advisor] for bars and clubs to go smokefree. However, the consensus among supporters of smokefree legislation was that "nobody [in government] is prepared to take the plunge" [union representative], for the reasons outlined below.

Tobacco control supporters recognized that "as with a lot of progress in tobacco control, it rarely happens suddenly; in fact, it never happens suddenly" [advocate], and acknowledged that incremental changes were important first steps in the early days of SHS advocacy. However, with bar and club workers remaining exposed to SHS, advocates were frustrated that "the timeframe that [the government] outline [s] is less ambitious than any of us ever contemplated back in 2000. We thought that maybe it would be a few years at most" [advocate].

A ministerial advisor acknowledged advocates' frustration, but explained that other government priorities make "things move more slowly than you'd like them to." Another advisor admitted that it was no longer a question of *if *smoking should be banned in pubs and clubs via legislation, but rather a question of *when *and *how *the government would act. However, this advisor suggested that, while it was clear that removing smoke from workplaces was the right health decision, SHS was a politically challenging issue that would not be fixed quickly. Instead, politicians and advisors indicated that incremental advances and compromises between various stakeholders are "the only way government can work" [political advisor] because of the government's need to take into account a variety of stakeholders. These interviewees also suggested that incremental change is "not unreasonable" [politician] because it "allows the community to go with you" [politician].

Because of the government's desire to balance various issues and stakeholders, politicians and advisors suggested that health advocates who are open to compromise and incremental progress will more likely be heard and received by the government. However, advocates felt challenged by this position because "if you just sit back and are nice to politicians all the time, does that really get you anywhere?...Somehow you've got to tell them that 'more needs to be done and that hasn't gone far enough"' [advocate] in protecting the public from SHS.

Advocates cautioned that incremental steps can slow progress towards comprehensive bans and contribute to the sense that an issue has been taken care of and, as a result, take some pressure off politicians to act more strongly. For example, several informants felt that compromises for smoke-free zones (such as no smoking within one metre of the drink service area) in bars and clubs were ways for the government to deal with SHS "politically" rather than "fundamentally" [advocate], by assuaging these industries but compromising health standards.

### Competition for the agenda with immediate issues

"I think that deep down they know it's the right thing to do, but that there are more immediate concerns, and I think that probably is what weighs more heavily on their minds." [advocate]

When attempting to explain the reasons why SHS legislation has failed to attract political urgency, most informants agreed that acute health issues, such as hospital bed shortages, unavoidably take precedence on the political agenda. Since political terms are only four years long, some bureaucrats suggested there is not much political investment in preventive health measures, such as smoking restrictions, because health improvements may not be evident for many years. A former health minister explained that "you cannot escape today by focusing on tomorrow." While championing tobacco control "was seen to be providing leadership and bringing some vision to the portfolio, from the negative view, it could be seen as taking time away from... [more] pressing issues."

Advocates proposed that one difficulty in raising the profile of SHS has been that "the idea of chronic exposure... is a difficult concept for people to take on board" [advocate]. While interviewees across the political spectrum acknowledged that the scientific evidence shows SHS is a health risk, tobacco control proponents indicated that they must still battle "an underlying sense of disbelief that [SHS] couldn't be as dangerous as everyone is saying" [advocate]. Because people do not often immediately become ill from SHS, advocates have found the dangers of SHS "a very difficult story to sell" [advocate] to both the public and politicians. Another advocate suggested that smoking was banned in workplaces and airplanes because people viewed involuntary exposure in a small environment as unacceptable; however, there is a sense that people choose to go to bars and, thus, are voluntarily exposed.

### Issue wear-out and sense that tobacco control has been "done"

"To use a transport analogy, it's a bit like putting a road through the mountains, and now we're down to curbing and guttering the highway. But putting the road through is the important thing." [politician]

Interviewees across the political spectrum also agreed that issue wear-out was inhibiting the attention given to SHS as a relevant and urgent issue. One advocate suggested that the biggest threat to advocates' success of achieving smoking bans in pubs and clubs was "a strong sense...that tobacco has been 'done"' by both members of the political and public health communities. Most informants indicated that politicians probably feel that they have already addressed the issue adequately, since most workers are now protected, and that they are content that "there are a few things to tidy up, but there are more important issues to worry about at the moment" [politician].

Tobacco control advocates also commented that issue wear-out has made it difficult to be heard by politicians or political advisors because the arguments and stakeholders surrounding the issue on both sides have remained the same. One advocate proposed that "unless you've got something new to say, [politicians] are not that interested." However, the challenge recognised by many interviewees, regardless of their affiliation, was that there was not much new to say on either side of the argument: health advocates cite the health evidence showing the harms of SHS, the economic studies from other jurisdictions showing that businesses are not harmed by bans and increasing public support, whereas the industry has attempted to keep SHS issues framed around economic losses and ideological arguments[2].

### Power of industry opposition

*"I just think there was a lot of political lack of will because the tobacco industry was still very active in its misinformation campaign, [and] that the hotels and others just kept saying 'Look, we'll go broke'*." [advocate]

Most interviewees acknowledged that the hospitality industry has played a significant role in the outcome of SHS legislation. Supporters of workplace smoking bans, including a representative from a restaurant industry association, expressed frustration with the level of influence that pubs and clubs have been able to wield in policy negotiations. These informants proposed that clubs and hotels have successfully avoided complete bans in their venues because they are "highly regarded" [politician] organisations that are "fairly important in the Australian context" [union representative]. Many important local identities tend to be hotel or club owners or associates and "that web of influence can be very important to politicians" [advocate]. A bureaucrat noted that, because of this influence, "there is a deeply entrenched view in government that there are no wins, and a lot of potential to lose, by tackling the bar situation."

Nearly all participants, including many of the politicians interviewed, reported their belief that the bar and club industries' political power was a reflection of financial donations to political parties. In contrast, the two political advisors were adamant that political donations played "zero" or "less of a role" than some advocates imagined. Instead, these political advisors suggested that the industries' influence with government is via the currency of job loss arguments – however spurious – because "if you make decisions that cost jobs, that's a very serious political issue" [political advisor].

Beyond these reasons, interviewees also suggested that the hotel and club industries have successfully promoted a pervasive sense in the community and among influential politicians that bars are somehow inherently "different" to other workplaces. Informants noted that bars are seen as a reasonable "last bastion" of smoking by many people, and that smoking in bars is "somehow emblematic of some form of Australian life that needed to be protected" [advocate]. Health bureaucrats, discouraged that their health-based policy advice is often ignored by politicians, reported that that because of industry lobbying the government was often more interested in a "comfort standard" [bureaucrat], such as ventilation systems and no-smoking zones that have been scientifically shown to be ineffective in reducing the health risk of tobacco smoke [32], rather than a comprehensive SHS ban.

### Evidence is outweighed by economic, ideological and anecdotal arguments

"It's not that they're not persuaded by the evidence, it's just that those other forces outweigh what they're trying to do." [bureaucrat]

Although all interviewees recognised SHS as a health risk, and most noted that governments would not act without a scientific and economic evidence base, they held different views regarding the extent to which this evidence can influence political decisions. Advocates stressed that evidence is "the foundation of your advocacy" [advocate]. However, politicians and political advisors indicated that evidence has limited power in governmental deliberations and noted that governments "can't make decisions based on the science alone" [advisor]. One political advisor went as far to say that "evidence" presented by any lobbyist is often viewed with circumspection because lobbyists tend to "spin" findings to suit their position.

Indeed, as suggested by some interviewees, if evidence were the only factor in political decisions, the NSW government would not have supported the partial restrictions supported by industry groups or have delayed comprehensive legislation for decades. Several interviewees proposed that the value of economic studies and scientific reports supporting smoking bans is diminished in political debates when countered by anecdotal economic and job loss arguments. A political advisor commented that it was one thing to talk to an industry lobbyist about projected economic losses, but it was "a very different thing to talk about a family member who has his entire business attached to a pub." A politician suggested that anecdotes and "folklore" stories of economic ruin have always carried more weight in political conversations than scientific studies and other forms of independent evidence. Interviewees also indicated that international evidence does not hold as much weight in political decision-making, as opponents can counter that Australia is "different," and highlighted the need for more Australian-specific studies to be conducted.

### The gambling factor

Participants also described how in recent years, the club and pub industries have framed their arguments around the threatened loss of gambling profit if smoking were banned in clubs and pubs (which have gaming machines), as gamblers tend often to be heavy smokers. Because of the "mutually beneficial income" [advocate] both the government and clubs get from gaming, some interviewees suggested that emphasising possible gambling profit loss might be "a convenient [argument] to distract attention away from the real issue – protecting people's health" [bureaucrat]. Several interviewees suggested that a problem facing advocates today is that there are no solid independent local studies relating to the impact of smoking bans specifically on gambling revenues in Australia. For example, because hotel-based gambling is not legal or widespread in most international jurisdictions that have already banned smoking in bars and clubs [33], the economic studies on which advocates have relied have never included the impact of smoking ban on gambling venues in pubs or hotels. Opposition groups, such as the AHA, have exploited this Australian "difference" and have argued that foreign studies of public support and positive economic impact are therefore irrelevant.

### What does the public believe?

"There isn't a visible constituency...that [resonates] with politicians, that gives them the impression, the belief, that there are real numbers attached to this issue for them electorally." [advocate]

Nearly all interviewees agreed that public opinion is an influential factor in political decisions; however, what was considered to constitute evidence of public opinion differed between interviewees. Supporters of smoking bans have "always been heartened by the results of public opinion polls" [advocate] that show majority popular support for bans, and expressed frustration that these appeared to have little impact on politicians. In contrast, politicians and advisors explained that political assessment of public sentiment is often "more of a gut feeling" [advisor]. One advisor considered that people who go to pubs and clubs in rural areas are a different constituency than in the city, and suggested that this electorate was not ready for smoking bans.

Despite documented public support for smoking bans, interviewees noted that policymakers appear to believe that those in favour of bans are not as passionate about their preferences as those opposed to bans, and that the intensity of the public's support with regard to bans in pubs and clubs remains in doubt. One advocate suggested that the only times politicians hear of smoking bans are from the "usual suspects": the same predictable handful of tobacco control advocates. Several informants reported their belief that the public seldom vocalizes its support of bans to parliamentary representatives. In the face of industry lobbying, an advocate suggested that if politicians believe that smoking bans are not a pressing issue for their constituents, they think "'Why should we incur all this angst from the AHA?"'

Interestingly, despite smokefree venues becoming the norm and politicians claiming that smoking bans are not an important issue for their constituents, informants intimated that policymakers still fear that there could be political "backlash" from select segments of the population. Some Labor Party members indicated that their members have a strong sense of "camaraderie with blokes in this country" [politician] who want to smoke in their "traditional" local pub, and that the government believes rural constituents are less likely to be supportive of smoking bans than city-dwellers [political advisor].

While a former health minister acknowledged that in hindsight there was no political fallout from banning smoking in workplaces and restaurants, he indicated that past reluctance on the part of both major parties to pass legislation in the early- and mid-1990s was due in large part to its sense that "there was political concern and fear that there could be a voter backlash in the event of the restriction of smoking" because many people smoked. However, previous concerns of voter-backlash regarding workplaces, public transport, and restaurants have proven to be unfounded with the public supporting the restrictions, as evidenced by high compliance rates and increasing public support [13,20,34].

### What will overcome the inertia of incrementalism?

"The law of inertia, isn't that what's going on? You need something more powerful in order to change the direction. The inertia right now is to do nothing." [union representative]

Similar to analysis of other tobacco control policy attempts [35-38], nearly all informants in this study believed that the challenge rests with advocates in NSW to "find a pathway...to make it politically advantageous" [advocate] for politicians to take action. Advocates and bureaucrats both acknowledged that the Health Department representatives had "laid everything on the table...[but] they can't influence the political process" [advocate] to progress SHS on the political agenda. Similarly, while minor party politicians and independent Members of Parliament had successfully pushed SHS onto the political agenda in the past by introducing bills and debate in Parliament, there was general agreement that only a bill introduced by the government would be accepted at this stage.

Advocates indicated that "the single most important thing... [is] that we remain committed and persistent" [advocate]. A political advisor agreed and stressed that although advocates may feel like they are "knocking their heads against a brick wall," they should "keep knocking because someone's [eventually] going to let you in."

Although no participant was able to conclusively state what they believed would be the "smoking gun" [union representative] that would overcome the inertia of incremental compromises in regards to pubs and clubs, they had a variety of suggestions of what tactics advocates should take to win the final endgame.

### Keep the issue focused on workers' rights and health to discredit industry arguments

Tobacco control supporters stressed the importance of keeping the issue of smoking bans centred on the concept of workers' rights and health protection. They suggested that the best way to counter the promotion of the "Australian narrative" [advocate] that smoking and drinking go together, is to frame smoking bans around the idea that "there is something fundamentally un-Australian about exposing workers to risks that we protect other workers from in every other situation" [advocate]. By highlighting SHS as a health inequity and making "the connection between the unpleasant experience" and the proven danger of SHS resonate with both the public and politicians [advocate], advocates may better influence community support and counter industry arguments about revenue loss [38,39].

### Mobilize a visible constituency

Most interviewees commented that one of the most powerful forces that would convince politicians to finally act would be the mobilization of a visible and diverse constituency demanding smoking bans in pubs and clubs. However, tobacco control supporters were sceptical about their ability to get the public impassioned about the issue because "we've reached a stalemate where places the empowered middle classes go are largely smokefree" [politician]. A union representative cautioned that "we're not going to necessarily going to get the public's perception to move all that much more than it already is," because, "to the extent to which you remove the problem for the majority," there are fewer people to view the issue as a problem.

### Use the media strategically

Nearly every participant commented that "the media have enormous power" [politician] in influencing both the public and political views about SHS. Media were seen as the "predominant" vehicle to educate the public [advocate], to "push politicians' buttons" [bureaucrat], and to contribute to changing social norms and expectations regarding the acceptability of passive smoking. Advocates and other tobacco control supporters indicated that they needed to strategically utilize the media by being available for public comment, calling into talk-back radio programs, writing letters to the editor, capitalizing on new evidence or events, and putting forward a vocal tobacco control position to counter-balance industry comments.

### Partnerships with other groups

Another strategy suggested by some informants to increase health advocates' profile with the public and gain more access to inside policy discussions, was partnerships with organisations, such as the labour unions, which the government sees as an important constituency. Advocates and the labour union representative noted that the formation of the "Smokefree '03" (now the "Smokefree Australia") campaign, comprising health charities, health advocacy groups and labour unions, broadened advocates' advocacy opportunities and access to politicians. For example, one advocate described how different organizations and individuals can play complimentary "good cop/bad cop" roles, with one organisation complimenting the government on its progress, and another taking a harder, critical stance in the media. Although some advocates cautioned that it can be challenging to present a united front or find agreement on issues, they believed a more cohesive strategy and acknowledgement of diverse allies could assist in advancing SHS on the political agenda.

### Demonstrations and grassroots campaigns

Interviewees described demonstrations and grassroots campaigns as another potentially influential strategy to put external pressure on the government. However, many participants said that the success of such strategies was dependent on the groups and individuals participating in the campaign, highlighting the importance of recognising each advocacy group's strengths and weaknesses in contributing to the SHS campaign. For example, some interviewees described how a hospitality labour union had effectively utilized grassroots lobbying to promote smokefree places – and had succeeded in influencing the Health Minister to include a ban on smoking at casino tables as part of the *Smokefree Environment Act 2000*. In contrast, while a community non-smokers advocacy group representative held the view that its rallies and letter-writing campaigns had given visibility to SHS issues, several other advocates cautioned that the group is perceived as "killjoys" [advocate] that lack credibility with policymakers and the community.

### More court cases needed

"If you get a couple of [litigation] cases where major pubs or clubs go down...you'll have the government...falling over themselves to introduce urgent legislation if necessary in Parliament." [politician]

Additional cases of litigation were another frequent suggestion about what would increase momentum on passive smoking legislation. Most participants said that previous court cases [40] had stimulated public debate, provided advocates with opportunity to increase media advocacy and allowed for tobacco control lobbying to be more favourably received by some politicians. Fear of litigation was viewed as a reason more clubs and pubs have voluntarily gone smoke-free [union representative], and the reason why the government went ahead with earlier SHS legislation [political advisor] [41].

The power of litigation to influence policy outcomes is supported by tobacco industry documents that show that the tobacco industry and its allies perceived early court cases such as major setbacks [28]. However, one advocate commented that at this stage court cases may not be "as threatening as they used to." For example, the most recent case in 2001, in which the NSW Supreme Court awarded damages to a non-smoking bar attendant who had developed throat cancer [42], did not "achieve the outcomes we were all hoping for" [advocate]. Although the case established a legal precedent and prompted supportive media articles that bars and clubs can be responsible for workplace-caused tobacco-related disease, it did not result in additional political action.

### Competition with other states

Many bureaucrats and politicians noted that NSW prides itself on leading other Australian states in government initiatives, and suggested that "one-upmanship" [bureaucrat] with other states might provide the stimulus for the NSW government to move faster. One politician noted that Australian states are like "dominos" and when one state takes the first step, the others will likely follow shortly after. For example, all Australian states followed the lead of the Australian Capital Territory (ACT) and then NSW by banning smoking in restaurants [43], and others have subsequently introduced laws to varying levels of comprehensiveness [44].

### Find champions in government

*"You've either got to have a lot of constituents knocking on the door saying I want something done, or you've got to have a politician with a fire in the belly who takes this problem and says 'We bloody need to deal with it*."' [politician]

In addition to putting external pressure on policymakers through the outside advocacy strategies described above, interviewees suggested that advocates must find a champion in government and convince him or her that supporting smoking bans is a good political decision. In the past, legislative attempts have only been successful when championed by a senior government politician. Several interviewees suggested that if the Premier or another high ranking politician had the courage to stand up to critics and back the issue, legislation to ban smoking in pubs and clubs could happen without much resistance.

## Discussion

In the past 20 years, the tobacco control movement in NSW has advocated successfully for considerable SHS legislation and regulation, and non-smoking indoor areas are now the public norm, enjoying wide support [13,20,34]. Yet the bar, club and casino industries continue to avoid total bans in their environments. In the three years since the interviews for this study were conducted, an agreement for comprehensive SHS legislation that includes bars has yet to be reached in NSW. Once again, the government has made concessions to industry, this time in the form of a definition of "enclosed spaces" that will allow smoking in areas that are substantially enclosed. Other Australian states (Tasmania and Queensland), Ireland, Norway, New Zealand, England and Scotland and several state governments in the USA and Canada have either implemented total indoor smoking bans, or legislated for their introduction.

The factors inhibiting the adoption of comprehensive restrictions, as described in this study, appear not to have dissipated. Interviewees' comments show that SHS restrictions in NSW have been contested and never been defined solely as being about achieving health outcomes, but have been delayed by several broad factors including: the continued influence of industry groups that have successfully opposed regulation; issue wear-out; and political perceptions that there is not a salient constituency demanding smoking be banned in bars and clubs.

### Industry influence

A major challenge for proponents of SHS regulation, as with most tobacco control endeavours, lies in the public benefits of regulation being perceived as diffuse, while the potential costs of regulation are concentrated on a vocal single interest group – the clubs and hotel industry [45]. In September 2004, in anticipation of the announcement that NSW would end smoking in pubs, the AHA sent its members a fax claiming their businesses would be "destroyed", that there would be "20% job loss" and that "61% of bars in Dublin will not survive much longer" [46]. despite reports from Norway, New Zealand and New York showing that the recent adoption of bans in these venues had minimal impact on business [47-49]. Although economic and ideological arguments against SHS legislation would appear to be both wrong and outdated, industry groups have succeeded in promoting a politically potent combination of these to the heart of NSW politics.

### Issue wear-out

Advocates operating in incrementalist political environments face the challenge that they may achieve only achieve a portion of their goals before their issue is "worn-out" and perceived to be "done" by politicians. Consonant with Nielsen's [39] observation of tobacco control in the United States, windows of opportunity [50] for creating tobacco control legislation may be closing in Australia as well. Issue wear-out may explain why other more immediate political concerns continually take precedence over SHS on the political agenda, why the public does not appear impassioned about the issue, and why SHS does not appear to politicians as a politically urgent problem that must be addressed.

### No visible constituency

Although strong and unambiguous public opinion can be a powerful motivator to persuade government to act [37,38,51], interviewees did not believe that politicians feel that the public demands legislative action in the way that often characterises other health issues which receive urgent political attention. As long as politicians do not perceive that a significant number of their constituents support smokefree laws as a significant problem that needs urgent attention, they will not feel the need to progress towards complete bans any faster [50,52-54].

As advocates suggested, because most politicians often only hear from "usual suspects" on this issue, advocates would be advised to mobilize other constituencies to vocalize support of smoking bans in bars and clubs. These new voices might include constituents in marginal political seats, bar workers, doctors, and other health workers, whose voices might resonate with politicians and provide personal stories to counter the anecdotes about economic ruin presented by opposition groups. If advocates are able to demonstrate overwhelming public support that spans the spectrum of constituents, politicians and their advisors will have to publicly defend why they continue to refuse to ban smoking in all workplaces, and will be less able to dismiss public opinion surveys as unrepresentative of the NSW population.

### Lessons for the endgame

Although advocates believed that past advancements towards smokefree areas were accomplished primarily through their groundwork strategies that would eventually be realised in the form of policies and laws, the support of a key legislator [38] was imperative in putting SHS on the political agenda both in 2000 (with restaurants and other public places) and in 2004 (with the latest in bars and clubs). No informant interviewed claimed to know anything precise about what influenced the Premier's apparently personal decision to take leadership on the issue and ban smoking in restaurants in 2000, alluding to his decision as a more or less inevitable response to the growing anti-smoking social environment in which he felt politically comfortable to act. Similarly, the entrance into politics of another champion in 2004 in the form of the newly appointed Minister for Cancer was undoubtedly critical to pushing bans in bars and clubs onto the political agenda [55,56]. As noted, the resulting legislation has been compromised in the face of industry lobbying, but the new law that will be implemented in July 2007 will see all fully enclosed rooms smokefree – another increment toward the final comprehensive total indoor smoking ban.

With the NSW government continuing to make concessions to industry demands, the strategies outlined by the participants in this study still have relevance to tobacco control advocates faced with similar incremental approaches. As with other studies, it appears that advocates in NSW have been the most successful when they utilize strategies that place outside pressure on policymakers, rather than trying to lobby from inside the political sphere, in which the industry excels [36,37,57].

In the past years, local advocates have capitalized on some of the tactics described in this study. An example was a 2005 media campaign in which an Irish bar patron mocks the NSW government for believing its residents are not ready for comprehensive bans when the Irish have been able to do it. This appropriated Australia's concern to not "lag behind" other countries [58]. By continuing to utilize their partnerships with groups that have visibility with the government and working on advancing smoking bans through the use of outside strategies – particularly media advocacy and the mobilization of additional voices – advocates can continue to create visible demand for smoking bans in bars and clubs and hold politicians accountable for the inequity of their exceptionalist policy of protecting all workers other than bar staff.

## Conclusion

Governments in the end stage of policy controls of SHS are often attracted to continuing drawn out incremental policy advances rather than risk confrontation with hospitality and tobacco interests. Advocates concerned to shorten the duration of this endgame must continue to insist that governments address the issue fundamentally by securing protection for all workers from SHS, rather than politically conceding to industry demands. Efforts are needed to both broaden and amplify community voices calling on governments to finish the job. Publicity to the growing number of state and national governments that have successfully implemented total bans over the past decade, as well as to continued public support, is likely to make incrementalism an increasingly unattractive political option.

## Competing interests

The author(s) declare that they have no competing interests.

## Authors' contributions

**KBJ **conducted the study, analysed the data and drafted the manuscript.

**SC **conceived of the study, supervised its conduct, and assisted in drafting the manuscript.

Both authors read and approved of the final manuscript.

## Pre-publication history

The pre-publication history for this paper can be accessed here:



## Figures and Tables

**Table 1:** Timeline of NSW government responses to secondhand smoke (SHS)

**Year**	**Actions**	**Notes**
**1970s**	Smoking banned on NSW buses and suburban trains, and in cinemas, theatres and public halls.	Smoking banned for comfort reasons.
**1983**	NSW *Occupational Safety and Health Act *1983	Law obligates all employers to ensure that their employees are not exposed to health or safety risks at work. Smoking not specified in Act.
	First NSW Parliamentary motion to ban smoking in workplaces and public places introduced as a private members' bill.	Parliament adjourned without addressing the motion.
**1992**	Smoking banned on NSW intercity trains	Smoking banned for comfort reasons.
	*Scholem vs NSW Department of Health *decided	NSW court handed down the world's first jury verdict on an employer's negligence in regard to passive smoking.
**1993**	*Occupational Health and Safety (Smoke-Free Indoor Air) Amendment Bill *proposed as a private members' bill	The Bill sought to amend the 1983 *Occupational Health and Safety Act *to include explicit statements to ban smoking in all indoor workplaces, including bars and restaurants, and included a 12 month phase-in period. The AHA lobbied extensively against the bill and it was not supported by government.
	NSW *Parliament House Smoke-Free Environment Act *passed	The Act banned smoking in the NSW Parliament House, but was ignored by some Members of Parliament.
**1995**	NSW *Passenger Transport Act 1995*	Amendments prohibited smoking on buses, ferries, taxis, and some private hire vehicles.
**1997**	NSW Passive Smoking Taskforce recommendations released	Taskforce convened by the NSW government consisted of NSW Department of Health bureaucrats, health advocacy groups, academics, and hospitality association representatives from restaurants, clubs and hotels. The taskforce recommended that the government ban smoking in all public places, including restaurants, bars and clubs. The only dissenting member was the AHA.
	NSW *Smoking Regulation Act 1997 *passed	The Act banned smoking in public places, but was virtually ineffective because bans were not to be implemented until five years after the Department of Health had established ventilation air quality standards.
**1999**	NSW *Rail Safety Regulation 1999*	Smoking banned on any NSW train or covered train platform.
**2000**	NSW *Smokefree Environment Act 2000 *passed	The Act banned smoking in enclosed public spaces, including restaurants and the dining areas of pubs and clubs. Although the original Bill included bars and clubs, these venues were exempt from the final act. When passed, the NSW *Smokefree Environment Act *was the most comprehensive SHS state law in Australia.
**2001**	*Sharp v Port Kembla Hotel & RSL Club *law suit verdict	A non-smoking bar attendant who developed throat cancer after 11 years of employment at a club was awarded $500,000 damages
**2003**	"Share the Air" voluntary agreement brokered with NSW club, pubs and hotels and NSW government	A Government and industry working group agreed that pubs and clubs will provide at least one smokefree bar and a 1.5 m no-smoking zone around bar areas. The NSW government promises to introduce formal legislation within a year.
**2004**	NSW *Smokefree Environment Amendment Act 2004 *passed	All enclosed areas of hotels, clubs and nightclubs that are open to the general public must be completely non-smoking by July 2007, as well as all areas of the Sydney casino, with the exception of private gaming rooms.
**2005**	First phase of NSW *Smokefree Environment Amendment Act 2004 *came into effect January 3, 2005	This phase replaced and formally legislated the voluntary "Share the Air" agreement. If a venue has: • More than one bar, then one bar should be non-smoking. • More than one of the same type of gambling area, then one of these should be non-smoking. • More than one recreational room that offers the same game or activity as another, then one of these should be non-smoking. Smoking is banned in all dining areas and at all counter and bar service areas.
	Second phase of NSW *Smokefree Environment Amendment Act 2004 *came into effect July 4, 2005	In a multi-room venue smoking allowed in a maximum of one room. The smoking room must not exceed 25% of the total combined area of the venue. If a venue consists of a single room, then smoking permitted in up to 25% of that area.
**2006**	NSW *Smokefree Environment Amendment Act 2006 *passed	The government amended the 2004 Act to adopt a definition of an "enclosed space" that will allow smoking in areas that are up to 75% enclosed in hotels, clubs and nightclubs. The Act is to be implemented in July 2007.
